# Association of Oral or Intravenous Vitamin C Supplementation with Mortality: A Systematic Review and Meta-Analysis

**DOI:** 10.3390/nu15081848

**Published:** 2023-04-12

**Authors:** Chongxi Xu, Tong Yi, Siwen Tan, Hui Xu, Yu Hu, Junpeng Ma, Jianguo Xu

**Affiliations:** 1 Department of Neurosurgery, West China Hospital, Sichuan University, No. 37, Guoxue Alley, Wuhou District, Chengdu 610000, China; 2 Department of Neurology, The Second People’s Hospital of Deyang City, No. 340 Minjiang West Road, Deyang 618000, China; 3 Outpatient Department, West China Hospital, Sichuan University, No. 37, Guoxue Alley, Wuhou District, Chengdu 610000, China; 4 Department of Neurosurgery, The Second People’s Hospital of Liangshan Yi, Autonomous Prefecture, Liangshan 615000, China

**Keywords:** vitamin C, COVID-19, sepsis, cancer, cardiac surgery

## Abstract

Mortality is the most clinically serious outcome, and its prevention remains a constant struggle. This study was to assess whether intravenous or oral vitamin C (Vit-C) therapy is related to reduced mortality in adults. Data from Medline, Embase, and the Cochrane Central Register databases were acquired from their inception to 26 October 2022. All randomized controlled trials (RCTs) involving intravenous or oral Vit-C against a placebo or no therapy for mortality were selected. The primary outcome was all-cause mortality. Secondary outcomes were sepsis, COVID-19, cardiac surgery, noncardiac surgery, cancer, and other mortalities. Forty-four trials with 26540 participants were selected. Although a substantial statistical difference was observed in all-cause mortality between the control and the Vit-C-supplemented groups (*p* = 0.009, RR 0.87, 95% CI 0.78 to 0.97, I^2^ = 36%), the result was not validated by sequential trial analysis. In the subgroup analysis, mortality was markedly reduced in Vit-C trials with the sepsis patients (*p* = 0.005, RR 0.74, 95% CI 0.59 to 0.91, I^2^ = 47%), and this result was confirmed by trial sequential analysis. In addition, a substantial statistical difference was revealed in COVID-19 patient mortality between the Vit-C monotherapy and the control groups (*p* = 0.03, RR 0.84, 95% CI 0.72 to 0.98, I^2^ = 0%). However, the trial sequential analysis suggested the need for more trials to confirm its efficacy. Overall, Vit-C monotherapy does decrease the risk of death by sepsis by 26%. To confirm Vit-C is associated with reduced COVID-19 mortality, additional clinical random control trials are required.

## 1. Background

Vitamin C (Vit-C) is an essential micronutrient required by the human body [1]. It serves as a potent antioxidant, preventing lipid peroxidation and protecting endothelial cells, making it significant in various diseases [2]. A population-based study of 384,282 patients found that intravenous Vit-C therapy was not associated with sepsis mortality [3]. In recent years, studies on Vit-C -based therapies, Vit-C + thiamine, and/or glucocorticoid cocktails have provided abundant data for future investigations. However, these studies provide inconsistent data on Vit-C therapy associated with reduced death rates. Several randomized controlled trials (RCTs) have revealed that neither Vit-C monotherapy [4] nor cocktails containing Vit-C [5] significantly reduce sepsis mortality. However, other RCTs determining the effectiveness of Vit-C supplementation in sepsis or septic shock patients, which resolves the shock more quickly and reduces the progression from sepsis to septic shock, thereby reducing mortality, and determining the length of hospital stay have also been performed [6,7].

Reducing mortality from advanced cancer has been a field of interest. Vit-C may be used as a tumor replacement therapy [8], supported by in vivo [9] and in vitro experiments [10]. A study performed in 1978 revealed that Vit-C therapy can notably prolong the survival rate of patients [11]. However, many RCTs prove the inconsistency of this fact [12,13] or neither approve nor disprove its effectiveness [14]. In terms of specific cancers, the results from one meta-analysis suggested that Vit-C intake can substantially decrease the mortality risk of breast cancer [15]. Intravenous infusions of ascorbic acid showed that it is effective when given combined with gemcitabine and radiation therapy for pancreatic cancer and adenocarcinoma that are locally metastasized [16]. Overall, the research on Vit-C and its efficiency against cancer is still of immense importance.

The coronavirus (COVID-19) pandemic has lasted over three years, and research on its pathophysiological mechanisms is ongoing [17]. Whether Vit-C a simple and easily available element and can be beneficial for COVID-19 treatment is still controversial. A retrospective study showed its positive effects in intensive care unit patients [18]; however, a systematic review and meta-analysis denied this effect [19]. As COVID-19 transmissibility increases, it is crucial to demonstrate the effectiveness of Vit-C, which is easy to obtain and particularly suitable for developing countries. Due to conflicting and limited evidence from previous studies, this investigation conducted a systematic review and meta-analysis of RCTs to assess the association between Vit-C therapy and mortality in patients with different disorders.

## 2. Methods

### 2.1. Eligibility Criteria

The trials were considered eligible if the patients were aged ≥18 years, if they were RCTs, if they compared intravenous or oral Vit-C at any concentration and compared against placebo or no treatment cohort, and if they reported all-cause mortality.

### 2.2. Exclusion Criteria

Trials were excluded if the patients were expecting or lactating, if Vit-C was used topically (e.g., colonoscopy bowel preparation [20] or cardioplegia solution containing Vit-C [21]), if the trials were case reports or observational study, if randomized controlled trials did not report mortality, or if they were not RCTs that studied the efficiency of Vit-C monotherapy (e.g., cocktails or nutrients containing Vit-C).

### 2.3. Information Sources and Search Strategy

The authors (TY) conducted a comprehensive search of multiple databases, including Medline (Ovid), Embase (Ovid), and the Cochrane Central Register of Controlled Trials (CENTRAL), from their inception up to 26 October 2022. They also searched ClinicalTrials.gov and the World Health Organization International Clinical Trials website. Language limitations were not applied during the search process. Supplemental Table S1 provides a detailed description of the search strategy used, including any filters and limitations that were applied to the search of all registers, databases, and websites. By conducting such a thorough and rigorous search, the authors were able to ensure that they included all relevant studies in their analysis and minimize the risk of publication bias.

### 2.4. Selection Process and Items

Duplicates were automatically removed using ENDNOTE 20 software. All the titles and abstracts were screened by two researchers (XCX and TSW) separately. Full-text articles assessed for eligibility were obtained, and those deemed eligible were further screened by reading the full text. Differences were settled by third review author MJP.

XCX and TSW independently extracted data from the selected trials using a standardized data extraction form. This form included information such as the year of publication, countries where the study was conducted, participant and intervention characteristics, funding sources, patient health condition, and mortality rates. In cases where a study had multiple experimental groups, data were extracted from the group that received Vit-C monotherapy, as well as the group that did not receive it. To obtain data from unpublished eligible trials registered in Clinical Trials.gov and the World Health Organization International Clinical Trials Registry Platform, the authors of these trials were contacted. Any disagreements between the two reviewers were resolved through consensus, by using a standardized data extraction form and resolving disagreements through consensus.

### 2.5. Outcomes

The primary outcome was all-cause mortality. Secondary outcomes were sepsis, COVID-19, cardiac surgery, noncardiac surgery, cancer, and other mortalities. All deaths of unknown causes reported in the study were classified as all-cause mortality according to their group.

### 2.6. Risk of Bias and Quality of Evidence

All the trials were selected according to the Cochrane Collaboration handbook for risk of bias, and their standard was separately evaluated by XCX and MJP via the revised Cochrane Collaboration risk of bias tool 2019 (ROB 2) [22]. For the selected trials, the process of randomization, intended intervention deviations, missing data outcome, assessment of the outcome, and reported results selection were evaluated. Based on the evaluation of the above 5 categories and 23 clauses, the trials were divided into high, unknown, and low bias risks. The evidence quality for outcomes was determined via the grading of recommendations assessment, development, and evaluation (GRADE) approach [23]. 

### 2.7. Effect Measures

Risk ratios (RR) and their related confidence intervals (CIs) of 95% were applied to determine all outcomes. A *p*-value of <0.05 was deemed statistically important. A random or fixed effects model was adopted according to the heterogeneity (I^2^ test) [24]. In the case of I^2^ ≥ 50%, a random effect model was utilized, or else a fixed effect model was used. Egger’s test, Harbord’s test, Begg’s test, and the AS-Thompsons test were used to evaluate the possibility of small study effects [25].

### 2.8. Data Synthesis

RevMan (version 5.4) and the meta package in R (version 4.2.2) were used to present and synthesize the results. The meta-analysis was evaluated by sequence trial analysis (TSA) (version 0.9.5.10) [26]. It was used to maintain 5% of the total risk type I error and 80% of the power. Originally it was expected that the relative risk for all-cause mortality was reduced by 10%, followed by a gradual reduction in the threshold until the optimal sample size increased more than the actual sample size.

Multiple subgroup assessments were performed according to the type of disease mortality (aforementioned mortalities), daily dose (≥4 g and <4 g), length of follow-up (<1 month and at least 1 month), latitude (≥40° and <40°), year of publication (before, in, and after 2015), country (Asian-Pacific, European, American, and international countries), the number of participants (≥100 and <100), and age (years) (≥60 and <60).

### 2.9. Sensitivity Analyses

To ensure the reliability and generalizability of the findings, sensitivity analyses were conducted using R software. These analyses involved deleting each trial one by one to assess the impact of its exclusion on the overall outcome. Furthermore, other sensitivity assessments were carried out, including the exclusion of trials with an uncertain or high bias risk, those with the largest heterogeneity within each subgroup, those conducted before the year 2000, those with follow-up times longer than 1 month, those with the largest number of participants, and those with high or unknown bias risk in various domains.

### 2.10. Patient and Public Involvement

No patients participated in the development of the study design or its implementation; moreover, no patient was asked about the results or explained. The results of this study can be disseminated to the public through the network or media.

## 3. Results

**Figure 1:**
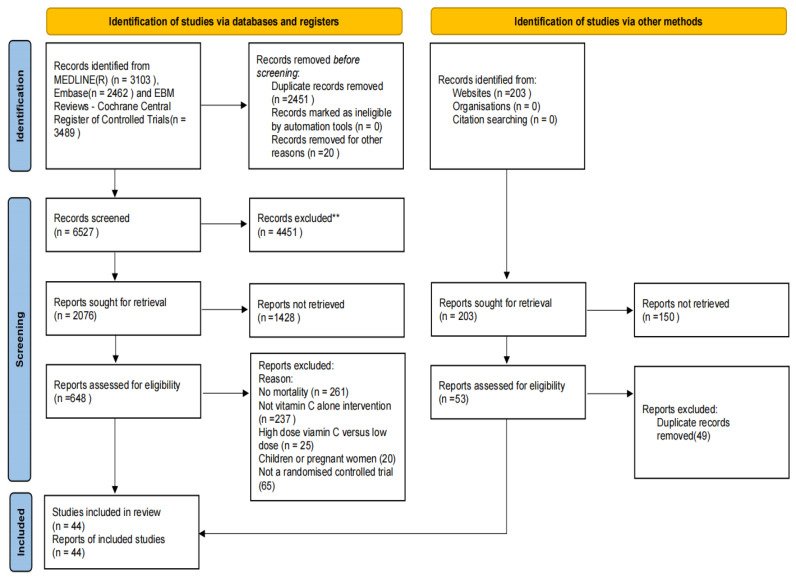
Search strategy and final included and excluded studies. ** Any excluded articles need to be double-checked by two people.

Nine thousand two hundred and fifty-seven records were searched, and 44 eligible trials were identified [4,5,7,12,13,27,28,29,30,31,32,33,34,35,36,37,38,39,40,41,42,43,44,45,46,47,48,49,50,51,52,53,54,55,56,57,58,59,60,61,62,63,64,65] (Figure 1). These 44 trials included 26540 participants. Fourteen of the trials involved 1918 patients with sepsis; seven involved 574 COVID-19 patients; four involved 22,491 cancer patients; seven had 954 patients who underwent cardiac surgery; five involved 150 patients with non-cardiac surgery; and seven other RCTs included 453 patients. A summary of these trials is shown in Table 1. Supplementary Table S2 presents the details of all 44 included trials which includes information on the number of participants in each trial, gender ratio, vitamin C supplementation dose and interval in the experimental group, and follow-up time for each trial. Additionally, 33 registered ongoing trials that met the criteria were also identified. However, they had incomplete or no reported results, and the summary of these details is shown in Supplemental Table S3.

Of the 44 eligible trials, 24 had a low risk of bias; 12 had an unclear risk of bias; and eight had a high risk of bias. The risk of bias in 44 trials is presented in Supplemental Figures S1 and S2. The evidence quality was high for the primary outcome (Supplemental Figure S3).

The validity and reliability of the meta-analysis results are important considerations in evaluating the quality and trustworthiness of the research. To this end, statistical tests were performed, including the Egger test (*p* = 0.0021), Begg test (*p* = 0.1753), Harbord test (*p* = 0.0042), and AS-Thompsons test (*p* = 0.0241), as well as a funnel plot analysis, to assess the presence of publication bias in the data. The results indicated that there was asymmetry in the data, which could be indicative of publication bias (see Supplemental Figure S4). However, to investigate the impact of this potential bias on the findings of the meta-analysis, we employed the trim-and-fill approach [66], This method involves estimating the number of missing studies and adjusting the results accordingly to create a more symmetrical funnel plot. The trim-and-fill analysis revealed that, despite the potential for publication bias, the results remained authentic and reliable (see Supplemental Figure S5).

No evidence was found related to the impact of small sample investigations on the results because of the robust combined effect of the remaining trials after deleting each trial (Supplemental Figure S6). After excluding studies with high or unknown risks of bias, trials conducted before 2000, trials with the highest number of participants, trials with a follow-up time exceeding 1 month, and trials with the highest level of heterogeneity in each subgroup, the results of the meta-analysis remained statistically significant (*p* < 0.05). Sensitivity analyses were conducted to assess the robustness of the findings regarding all-cause mortality, and the results are presented in Supplemental Table S4. Therefore, the meta-analysis’s sensitivity analyses suggest that the results are reliable and robust.

Of the 44 trials, all-cause mortality was reported in RCTs. There was a substantial statistical difference in all-cause mortality between the Vit-C supplemented and the control groups (*p* = 0.009, RR 0.87, 95% CI 0.78 to 0.97, I^2^ = 36%; Supplemental Figure S7).

According to the TSA analysis, the available data on all-cause mortality did not meet the criteria for a significant reduction in relative risk of 10% or 7.5%. As a result, the effectiveness of these results cannot be confirmed at this time. This finding is supported by the Supplemental Figures S8 and S9, which demonstrate that the quantity of information available for this outcome did not meet the required threshold for statistical significance. Meta-regressions indicated no link between the dose of Vit-C supplementation and all-cause mortality (*p* > 0.05).

**Figure 2:**
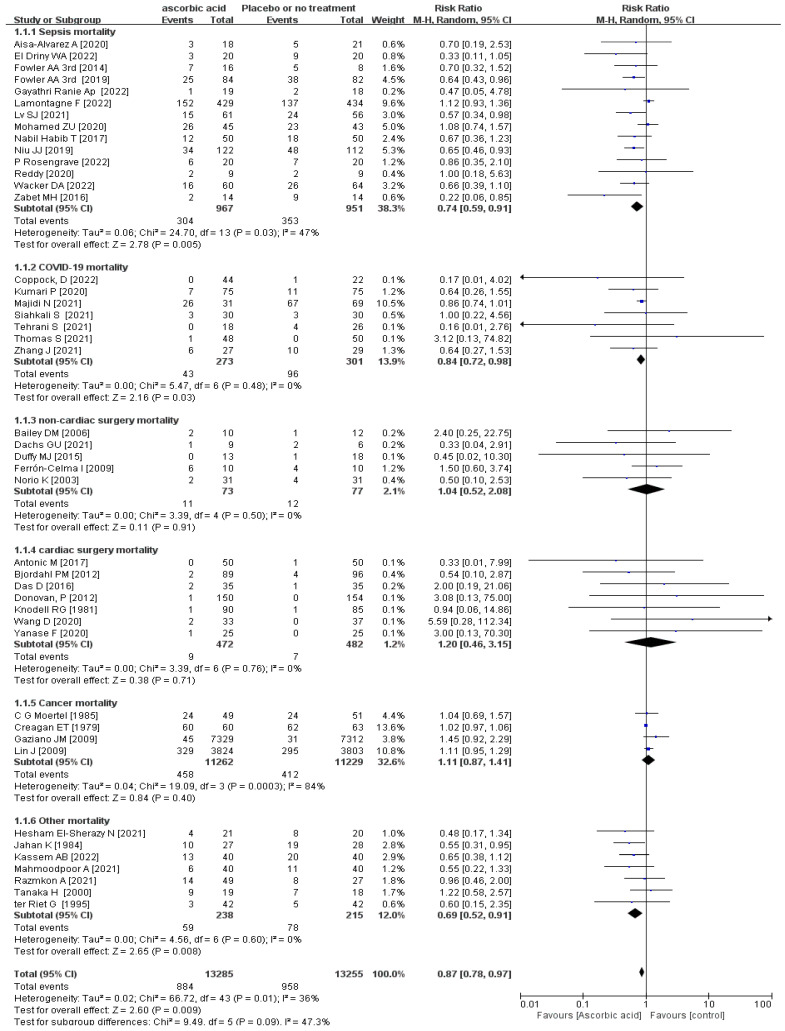
Forest plot of sepsis mortality, cardiac surgery mortality, non-cardiac surgery mortality, COVID-19 mortality, cancer mortality, and other mortality of trials evaluating vitamin C supplementation [4,5,7,12,13,27,28,29,30,31,32,33,34,35,36,37,38,39,40,41,42,43,44,45,46,47,48,50,51,52,53,54,55,56,57,58,59,60,61,62,63,64,65].

**Figure 3:**
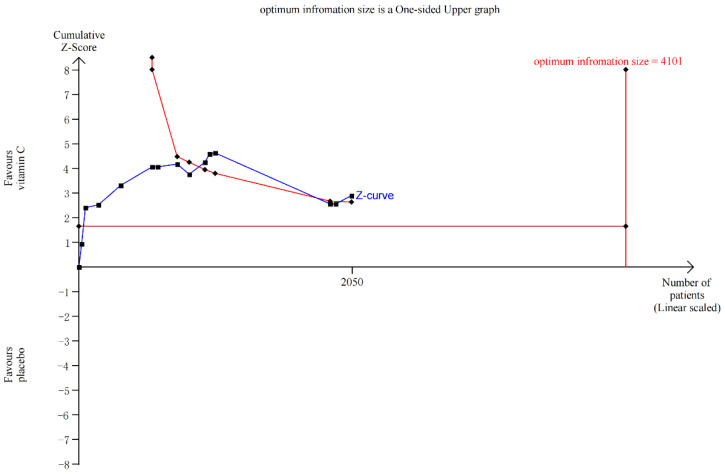
Trial sequential analysis for sepsis mortality, relative risk reduction = 10%.

The subgroup analyses revealed a statistically substantial difference in sepsis-associated mortality between the Vit-C -supplemented and control groups (*p* = 0.005, RR 0.74, 95% CI 0.59 to 0.91, I^2^ = 47%; Figure 2), COVID-19 (*p* = 0.03, RR 0.84, 95% CI 0.72 to 0.98, I^2^ = 0%; Figure 2), and other mortality (*p* = 0.008, RR 0.69, 95% CI 0.52 to 0.91, I^2^ = 0%; Figure 2). Other mortality included RCTs about patients with severe head injury [41], severe pneumonia [27], pressure ulcers [28], critical illness [56], severe burns [60], transfusion-related acute lung injury [59], and tetanus [30]. Although in TSA, the information size of COVID-19 and sepsis patients’ mortality did not meet the 10% relative risk reduction, in TSA model of sepsis-associated mortality cases, at least 4101 participants are required to obtain effective results; however, the blue cumulative z-curve was constructed using a random-effects model, and it crossed the traditional boundary value and TSA boundary value, which means that although the accumulated information did not reach the expected value, it obtained a positive conclusion in advance. So, the results of sepsis were confirmed in advance (Figure 3), while for that of COVID-19, the blue cumulative z-curve crossed the traditional boundary value; however, it did not cross the TSA boundary value, which means that false positives may have been obtained and more trials need to be included to confirm efficacy (Supplemental Figure S10). Our results showed no significant impact of vitamin C supplementation on mortality rates in patients undergoing cardiac surgery, non-cardiac surgery, or cancer (*p* > 0.05) (Figure 2).

Further results from the subgroup analysis of the effect of vitamin C on all-cause mortality are presented in Table 2. The analysis suggests that trials with more than 100 participants showed significant beneficial effects of vitamin C supplementation on all-cause mortality.

A diversity-adjusted information size of 4101 patients was calculated based on an anticipated relative risk reduction of 10% (event proportion of 37% in the control arm, α = 0.05 (one-sided), β = 0.20 (power 80%)). The blue cumulative z-curve was constructed using a random-effects model, and it crossed the traditional boundary value and TSA boundary value, which means that although the accumulated information did not reach the expected value, it obtained a positive conclusion in advance.

## 4. Discussion

### 4.1. Principal Findings

The quality of all the included 44 RCTs and 26,540 participants in this meta-analysis is high and showed a statistically substantial difference in all-cause mortality between the Vit-C supplemented and the control groups. However, it may be “Potentially spurious evidence of effect” from the TSA results and from the TSA model calculation results; at least 57,356 participants are required to obtain effective results. Therefore, to confirm its efficiency more trials are required. In subgroup analysis, the quality of trials that reported the sepsis mortality was high and had a statistically substantial difference between the Vit-C supplemented and control groups. Additionally, in TSA, although the information size of sepsis mortality did not meet the 10% relative risk reduction criteria, the cumulative z-curve surpassed the monitoring boundaries, which revealed “Firm evidence of effect”. A notable statistical difference in COVID-19 mortality between the Vit-C -supplemented and control groups was observed. However, the quality of trials that reported COVID-19 mortality was moderate, and the cumulative z-curve did not surpass the monitoring boundaries which revealed a result of “Potentially spurious evidence of effect” from TSA analysis and its model suggests that at least 1668 more participants are needed to get valid results.

### 4.2. Strengths and Limitations

This meta-analysis represents a pioneering effort to investigate the association between vitamin C and all-cause mortality, and we strictly followed an a priori protocol. The quality of evidence from the trials was rigorously assessed using the GRADE approach, and the trials that reported all-cause mortality were of high quality. Our subgroup analysis was comprehensive, aiming to shed light on the impact of vitamin C on mortality rates across diverse settings. The sensitivity analyses conducted in this study offer a thorough assessment of its robustness, consistent with the high-quality scientific standards expected in research. This rigorous approach helps to ensure the reliability of the study’s findings and sheds light on potential sources of heterogeneity and bias.

However, it is worth noting that inconsistencies in findings may arise due to differences in patient characteristics. For instance, the survival rate of patients with advanced cancers may be very low, irrespective of vitamin C supplementation. Moreover, multiple randomized controlled trials of vitamin C therapy did not report mortality outcomes. Many trials on other vitamins, for example, trials of vitamin D, report different forms or sources and found that they do have different effects on the final outcome; however, most of the randomized controlled trials did not provide specific sources and forms of vitamin C; hence, this information was not considered in the analyses. Future trials should detail this information in their papers. In addition, most of the trials only reported the dose of Vit-C, and serum circulating levels were not assessed thus, the dose–response relationship was unclear. 

### 4.3. Comparisons with Other Studies

There have been no systematic reviews of Vit-C and all-cause mortality. Many ascorbic acid-related meta-analyses are based on specific issues or diseases. The trials of Vit-C monotherapy for sepsis are very few, and most of them are cocktails containing Vit-C (e.g., ascorbic acid, corticosteroids, and thiamine). However, these results are inconsistent. A meta-analysis of 33 RCTs involving 9898 patients indicated no strong evidence that supported the routine ascorbic acid therapy in reducing mortality [67]. Nevertheless, a meta-analysis of 10 RCTs and 1400 participants [68] indicated that Vit-C reduces short-term mortality in sepsis patients; however, it still seems to be controversial. From this analysis, in the 14 RCTs with 1918 sepsis patients, Vit-C monotherapy was notably associated with mortality (*p* = 0.005, RR 0.74, 95% CI 0.59 to 0.91, I^2^ = 47%). In TSA, although the information size of sepsis mortality did not meet the 10% reduced relative risk criteria, the curve crosses both the traditional and TSA boundary values, revealing “firm evidence of effect” [26].

A meta-analysis of six RCTs with 572 patients indicated no benefit of Vit-C administration on COVID-19 patients [19], whereas another meta-analysis with 19 trials recommended Vit-C therapy as it reduced the mortality and length of hospital stay [69]. Although some of these studies included a cocktail with Vit-C (e.g., high-dose Vit-C, melatonin, and zinc [70]), the results were controversial. Stemming from the data of this meta-analysis, seven trials of RCTs revealed a notable statistical difference in COVID-19-associated mortality between the Vit-C monotherapy and the control groups (*p* = 0.03, RR 0.84, 95% CI 0.72 to 0.98, I^2^ = 0%). However, the TSA revealed a false positive result, indicating a need for more trials to confirm the efficacy.

Previous studies have shown the protective effect of Vit-C against contrast-mediated nephropathy before cardiac surgery [71] and preventing atrial fibrillation after the cardiac surgery [29,72]. Based on the previous literature, it was initially hypothesized that Vit-C could reduce mortality in patients undergoing cardiac surgery by preventing atrial fibrillation and improving endothelial function; however, the results showed no such evidence. Furthermore, no association between Vit-C and cancer-mortality was observed, although no evidence suggested a causal link between circulating Vit-C concentration and a variety of cancers; in certain types, however, it may present different values, for example, increased lung cancer incidence in women [73], decreased likelihood of recurrence of breast cancer [74], or enhanced tumor radiosensitization in pancreatic cancer [16]. Therefore, we believe that although vitamin C cannot reduce the overall mortality rate of cancer patients, whether it has a beneficial effect on different types of cancer should be analyzed in detail and depth.

### 4.4. Implications

Mortality is the most clinically serious outcome, and its prevention remains a constant struggle. This study involved patients with all conditions. However, the study size did not meet the 10% optimum relative risk reduction criteria and was unable to prove that Vit-C therapy has a clinically efficient impact on all-cause mortality. Similarly, this investigation fails to prove that Vit-C can reduce mortality in COVID-19 patients. However, the value of vitamin C in the above situations cannot be ignored. Importantly, Vit-C therapy does reduce sepsis mortality by 26%. Therefore, this investigation supports Vit-C supplementation in sepsis patients, and a more targeted intervention basis on Vit-C (e.g., a cocktail) seems appropriate. We suggest considering the addition of a combination of vitamin C and hydrocortisone to standard treatment as a potential treatment option for sepsis management. This treatment approach has shown promise in some studies and may be worth further investigation. This meta-analysis also found the role of vitamin C in other diseases, such as pressure ulcers, severe pneumonia, severe burns, and transfusion-related acute lung injury. In these trials, vitamin C effectively reduced mortality; however, their participants were minimal and could not effectively support the research results. Nevertheless, these findings are still meaningful and provide more research directions for the relationship between vitamin C and various diseases.

Currently, 33 trials with 5215 participants who meet the criteria have been registered and are ongoing. Among them, seven trials were related to sepsis; six were related to COVID-19; five were related to cancer; three were related to severe pneumonia; three were related to liver cirrhosis; and the remaining nine trials include kidney injury, acute pancreatitis, dengue fever, brain trauma, cardiac surgery, etc. These trials will further supplement our data. In particular, the correlation of Vit-C supplementation with all-cause mortality and COVID-19 mortality will be further clarified. 

## 5. Conclusions

Vitamin C therapy may reduce all-cause mortality when compared with a placebo or no treatment; however, more RCTs to confirm its efficiency are needed. Nevertheless, Vit-C monotherapy reduced the risk of death by sepsis by 26%. Additional multiple clinical RCTs are needed to assess whether Vit-C therapy is linked with lower COVID-19-related mortality.

## Figures and Tables

**Table 1:** Summary characteristics of included studies.

Eligible Studies:	No of Trials (No of Participants)
Total No of trials (No of participants)	44 (28,540)
Median follow-up (days)	150 (days)
Follow-up less than 1 month	35 (3730)
Median No of participants	603 (40–121)
Total No of deaths	1842
Median% male	71%
Median age (years)	64.3
Country	
European	5 (257)
American	13 (24,496)
Asian-Pacific	7 (604)
International country	19 (1183)
Dose	
Dose ≥ 4 g daily	22 (16,779)
Dose < 4 g daily	22 (9861)

**Table 2:** Subgroup analysis of the effect of vitamin C on all-cause mortality.

Subgroup Title	No of Trials	No of Participants	I^2^ (%)	Risk Ratio (95% CI)	*p* for Interaction
Overall	44	26,540	31	0.84 (0.75–0.95)	0.03
No of participants:					
≥100	16	25,109	0	0.89 (0.77–1.04)	<0.01
<100	28	1431	51	0.76 (0.62–0.93)	0.62
Year of publication					
In or after 2015	30	3081	18	0.75 (0.65–0.88)	0.20
Before 2015	14	23,459	1	0.84 (0.75–0.95)	0.44
Follow-up					
At least 30 days	9	24,686	0	0.84 (0.75–0.95)	0.80
Less than 30 days	35	3730	16	0.74 (0.63–0.86)	0.21
Age (years):					
≥60	19	24,434	22	1.01 (0.92–1.10)	0.18
<60	25	2106	0	0.71 (0.60–0.83)	0.82
Daily dose(g)					
≥4	22	16,779	32	0.84 (0.69–1.02)	0.07
<4	22	5378	31	0.84 (0.71–0.99)	0.08
Latitude:					
≥40	17	24,472	31	1.02 (0.99–1.05)	0.30
<40	27	2068	0	0.74 (0.64–0.86)	0.57
country					
Asian-Pacific	7	604	0	1.02 (0.99–1.05)	0.46
International countries	19	1183	0	0.73 (0.60–0.88)	0.51
American	13	24,496	25	1.02 (0.99–1.05)	0.19
European	5	257	0	1.10 (0.55–2.17)	0.64

## Data Availability

The original contributions presented in the study are included in the article/supplementary material, further inquiries can be directed to the corresponding author.

## Supplementary Materials

The following are available online at https://www.mdpi.com/article/10.3390/nu15081848/s1, Supplemental Figure S1. Risk of bias summary: each risk of bias item for each included trial. Supplemental Figure S2. Risk of bias graph: each risk of bias item is presented as percentages across all included trials. Supplemental Figure S3. Summary of Findings and Strength of Evidence in Studies. Supplemental Figure S4. Funnel plot of all-cause mortality (classified by sepsis, cardiac surgery, non-cardiac surgery, cancers, COVID-19, and others). Supplemental Figure S5. Funnel plot of all-cause mortality after the trim-and-fill method. Supplemental Figure S6. Sensitivity analysis was conducted by deleting the trials one by one. Supplemental Figure S7. Forest plot of all-cause mortality of trials evaluating vitamin C supplementation. Supplemental Figure S8. Trial sequential analysis for all-cause mortality, relative risk reduction = 10%. A diversity-adjusted information size of 57,356 patients was calculated based on an anticipated relative risk reduction of 10% (event proportion of 7.2% in the control arm, α = 0.05 (one-sided), β = 0.20 (power 80%)). The blue cumulative z-curve was constructed using a random-effects model and did not cross the TSA boundary value, which means that false positives may have been obtained and more trials need to be included to confirm efficacy. Supplemental Figure S9. Trial sequential analysis for all-cause mortality, relative risk reduction = 7.5%. A diversity-adjusted information size of 103207 patients was calculated based on an anticipated relative risk reduction of 7.5% (event proportion of 7.2% in the control arm, α = 0.05 (one-sided), β = 0.20 (power 80%)). The blue cumulative z-curve was constructed using a random-effects model and did not cross the TSA boundary value, which means that false positives may have been obtained and more trials need to be included to confirm efficacy. Supplemental Figure S10. Trial sequential analysis for COVID-19 mortality, relative risk reduction =10%. A diversity-adjusted information size of 2242 patients was calculated based on an anticipated relative risk reduction of 7.5% (event proportion of 31.9% in the control arm, α = 0.05 (one-sided), β = 0.20 (power 80%)). The blue cumulative z-curve was constructed using a random-effects model and did not cross the boundary TSA boundary value, which means that false positives may have been obtained and more trials need to be included to confirm efficacy. Supplemental Table S1 search strategy. Supplemental Table S2. details of Study characteristics. Supplemental Table S3. Ongoing trials of vitamin C supplementation on mortality. Supplemental Table S4. sensitivity analyses.

## Study Registration

Prospero registration number CRD42022369724.
