# Supplementary material for: Association of Oral or Intravenous Vitamin C Supplementation with Mortality: A Systematic Review and Meta-Analysis
Source: Nutrients. 2023 Apr 12;15(8):1848. doi: 10.3390/nu15081848 (PMC10146309; doi:10.3390/nu15081848)
Supplement: Supplementary file 1 [file nutrients-15-01848-s001.zip › supplemental Table S1.pdf]

Supplemental Table S1 search strategy

MEDLINE(R)		
1	**exp Ascorbic Acid/ad, ae, pd, tu [Administration & Dosage, Adverse Effects, Pharmacology, Therapeutic Use]**	21370
2	**((Acid, Ascorbic or L-Ascorbic Acid or Acid, L-Ascorbic Or L Ascorbic Acid or Vitamin C Or Hybrin Or Magnorbin or Sodium Ascorbate Or Ascorbate, Sodium Or Ascorbic Acid, Monosodium Salt Or Ferrous Ascorbate Or Ascorbate, Ferrous Or Magnesium Ascorbate Or Ascorbate, Magnesium Or Magnesium di-L-Ascorbate Or Magnesium di L Ascorbate Or di-L-Ascorbate, Magnesium Or Magnesium Ascorbicum)adj5 (supplement* or therap* or treat* or prevent* or daily or receiv* or regimen or dose? or oral* or intramuscular or inject*)).mp**	6261
3	**randomized controlled trial.pt.**	579481
4	**Controlled clinical trial.pt.**	95081
5	**randomized.ab.**	580199
6	**placebo.ab.**	232729
7	**drug therapy.fs.**	2541363
8	**randomly.ab.**	394124
9	**trial.ab.**	621299
10	**groups.ab.**	2425687
11	**3 or 4 or 5 or 6 or 7or 8 or 9 or 10**	5494228
12	**exp animals/ not humans.sh.**	5058780
13	**11 not 12**	4788332
14	**(1 or 2) and 13**	6261
15	**limit 14 to "all adult (19 plus years)"**	3103
**Embase**		
1	**exp ascorbic acid/ae, ct, ad, cb, cm, cr, do, dt, ce, ci, dl, du, ig, ly, im, na, os, tl, ur, iv, po, li, tp, td [Adverse Drug Reaction, Clinical Trial, Drug Administration, Drug Combination, Drug Comparison, Drug Concentration, Drug Dose, Drug Therapy, Intracerebral Drug Administration, Intracisternal Drug Administration, Intradermal Drug Administration, Intraduodenal Drug Administration, Intragastric Drug Administration, Intralymphatic Drug Administration, Intramuscular Drug Administration, Intranasal Drug Administration, Intraosseous Drug Administration, Intrathecal Drug Administration, Intraurethral Drug Administration, Intravenous Drug Administration, Oral Drug Administration, Sublingual Drug Administration, Topical Drug**	25932

	**Administration, Transdermal Drug Administration]**	
**2**	**((Acid, Ascorbic or L-Ascorbic Acid or Acid, L-Ascorbic Or L Ascorbic Acid or Vitamin C Or Hybrin Or Magnorbin or Sodium Ascorbate Or Ascorbate, Sodium Or Ascorbic Acid, Monosodium Salt Or Ferrous Ascorbate Or Ascorbate, Ferrous Or Magnesium Ascorbate Or Ascorbate, Magnesium Or Magnesium di-L-Ascorbate Or Magnesium di L Ascorbate Or di-L-Ascorbate, Magnesium Or Magnesium Ascorbicum)adj5 (supplement* or therap* or treat* or prevent* or daily or receiv* or regimen or dose? or oral* or intramuscular or inject*)).mp**	7546
**3**	**randomized controlled trial/**	733606
**4**	**crossover procedure/**	71825
**5**	**double blind procedure/**	199982
**6**	**single blind procedure/**	48019
**7**	**(random* or factorial* or crossover* or placebo* or assign* or allocat* or volunteer* or (doubl* adj5 blind*) or (singl* adj5 blind*)).mp.**	3036224
**8**	**or/3-7**	3036224
**9**	**exp animal/**	29258744
**10**	**human/**	24095686
**11**	**9 not 10**	5163058
**12**	**8 not 11**	2751553
**13**	**(1 or 2) and 12**	5754
**14**	**limit 13 to (adult <18 to 64 years> or aged <65+ years>)**	2426

EBM Reviews - Cochrane Central Register of Controlled Trials

**1**	**exp Ascorbic Acid/**	2357
**2**	**((Acid, Ascorbic or L-Ascorbic Acid or Acid, L-Ascorbic Or L Ascorbic Acid or Vitamin C Or Hybrin Or Magnorbin or Sodium Ascorbate Or Ascorbate, Sodium Or Ascorbic Acid, Monosodium Salt Or Ferrous Ascorbate Or Ascorbate, Ferrous Or Magnesium Ascorbate Or Ascorbate, Magnesium Or Magnesium di-L-Ascorbate Or Magnesium di L Ascorbate Or di-L-Ascorbate, Magnesium Or Magnesium Ascorbicum)adj5 (supplement* or therap* or treat* or prevent* or daily or receiv* or regimen or dose? or oral* or intramuscular or inject*)).mp**	1937
**3**	**1 or 2**	3489
