# Supplementary material for: Association of Oral or Intravenous Vitamin C Supplementation with Mortality: A Systematic Review and Meta-Analysis
Source: Nutrients. 2023 Apr 12;15(8):1848. doi: 10.3390/nu15081848 (PMC10146309; doi:10.3390/nu15081848)
Supplement: Supplementary file 1 [file nutrients-15-01848-s001.zip › supplemental Table S2.pdf]

Supplemental Table S2 details of Study characteristics

Trial	Participants (vitamin C/no vitamin C)	Mean age	male	Intervention	Control	Primary outcome	Follow-up
Yanase F 2020	25/25	65.5	76%	high-dose intravenous vitamin C (1500 mg every 6 hours)	placebo	time to resolution of postoperative vasoplegia.	8 days
Wang D 2020	33/37	57	38.6%	intravenous vitamin C 1 g	saline	postoperative pulmonary complications	15 days
Das D 2016	35/35	46.7	NR	oral Vitamin C (500 mg) twice daily	placebo	cortisol secretion	30 days
Antonic M 2017	52/53	64.81	78%	2g 24h before and after surgery. 1 g twice a day for five days after the surgery	placebo	an episode of Atrial fibrillation or flutter lasting>10 min	7 days
Bjordahl PM 2012	89/96	63	67%	2g before surgery and 1 g twice daily after (5 days)	placebo capsule	the presence of postoperative atrial fibrillation or atrial flutter for 10 minutes	20 days
Knodell RG 1981	6/8	57.95	71%	800 mg 4 times daily for 2 days before surgery and for 2 wk postoperatively	lactose placebo	posttransfusion hepatitis	6 month
Donovan, P 2012	150/140	61	76%	2 g, PO, the night before surgery, 1g, PO bid,<br>the day of surgery to postop 5th day	Beta Blockers and Amiodarone	Postoperative Atrial fibrillation	30 days
Creagan ET 1979	60/63	NR	61.8%	10 g per day	placebo	survival	At least 63 weeks
C G Moertel	51/49	NR	57%	10 g daily	placebo	survival	1 year

1985							
Lin J 2009	3803/3824	60.4	women	500 mg daily	placebo	Cancer death	5 years
Gaziano JM 2009	7329/7312	NR	man	500-mg daily	placebo	prostate and total cancer events	8.0 years
Duffy MJ 2015	13/18	71.8	90%	intravenous 2 g	placebo	endothelial dysfunction	10 days
Dachs GU 2021	10/10	66.45	55%	intravenous 1 g/kg QD for 4 days	No vitamin c	Protein markers of tumor hypoxia or DNA damage	30 days
Ferrón-Celma I 2009	9/6	73	80%	450 mg/d 6 days	placebo	Neutrophil count and percentage	6 days
Bailey DM 2006	10/12	68	NR	intravenous 2 g before surgery	Placebo	oxidative lipid damage	hospital stays
Razmkon A 2021	49/27	30	86.8%	10 g IV first day and fourth day, 4 g/d the other 3 days; 500 mg/d IV for 7 days of another group	placebo	Glasgow Outcome Scale (GOS) scores	6 months
Mahmoodpoor A 2021	40/40	54.59	57%	60 mg/kg/ day	saline	Acute Physiology and Chronic Health Evaluation	28 days
Hesham El-Sherazy N 2021	21/20	43.6	68%	orally at a dose of two grams twice daily	saline	vancomycin associated nephrotoxicity	28 days
Norio K 2003	31/31	NR	NR	1g IV QD	conventional treatments	mortality	4 weeks
ter Riet G 1995	43/45	NR	NR	500mg twice daily	placebo	Wound status	90 days
Jahan K 1984	27/28	NR	NR	1g IV QD	NR	mortality	NR
Tanaka H 2000	19/18	44.5	67.5%	66mg/kg per hour in 1 day	placebo	resuscitation fluid volume requirements	7 days
Kassem AB 2022	40/40	49.9	56%	2.5 g/6 h intravenous for 96h	placebo	Serum inflammatory level	28 days
Tehrani S	18/26	59.5	59%	2g q6h for 5	standard	oxygen saturation	6 days

2021				days	treatmen t		
Thomas S 2021	48/50	43.8	35%	8000 mg over 2-3 times per day	standard treatmen t	number of days required to reach a 50% reduction in symptoms,	28 days
Coppock, D 2022	44/22	60.5	NR	0.6 g/kg day one and 0.9 g/kg 5 days	Standar d of Care	composite outcome	28 days
Zhang J 2021	27/29	66.5	66.1%	12 g every 12 h for 7 days	placebo	clinical improvement within 72 h	28 days
Siahkali S 2021	30/30	59.2	50%	1.5g iv q6h 5 days	standard treatmen t	composite outcome	9 days
Majidi N 2021	31/69	60.12	60%	500 mg QD 14d ays	nutrition	Biochemical and Clinical Indices	14 days
Kumari P 2020	75/75	52..5	NR	50 mg/kg/day (IV)	standard therapy	composite outcome	14 days
Nabil Habib T 2017	50/50	42.2%	58%	1.5 g q6h iv	control	Acute Physiology and Chronic Health Evaluation II	21 days
Lv SJ 2021	61/56	59.4	50.4%	3 g iv twice a day	basic therapy	28-day mortality.	28 days
Zabet MH 2016	14/14	63.9	75%	25 mg/kg iv q6h For 72h	Control	Vasopressor dose and duration	29 days
Gayathri Ranie Ap 2022	20/20	NR	NR	2g q8h iv 5 days	Control	Sepsis Related Organ Failure Assessment	6 days
Fowler AA 3rd 2019	84/83	55.5	53.9%	50 mg/kg iv	placebo	modified Sequential Organ Failure Assessment score	28 days
El Driny WA 2022	20/20	52.5	52.5%	1.5 g/6 h iv for days	100 mg	sequential organ failure assessment	28 days
Wacker DA 2022	60/64	70.9	50.8%	250mg/h for 96 h	placebo	all-cause 28-day mortality	28 days
P Rosengrave 2022	20/20	68.5	67.5%	25 mg/kg pump	placebo	vasopressor requirements	96 days

Lamontagne F 2022	423/433	65.1	62%	50mg/kg q6h iv for 96h	placebo	death or persistent organ dysfunction	28 days
Reddy 2020	9/9	56	42.8%	1.5 g IV q6h+ hydrocortisone	hydrocortisone alone	time from initiation of therapy to shock reversal	NR
Fowler AA 3rd 2014	16/8	NR	54.2%	50 mg/kg/24 h group and 200 mg/kg/24h group	Placebo	ascorbic acid safety and tolerability	28 days
Aisa-Alvarez A 2020	18/21	69	41%	1 mg Vit C tablets daily	control	organ dysfunction	5 days
Mohamed ZU 2020	45/43	59.03	71.6%	1.5 g every 6 hours plus thiamine and hydrocortisone	thiamine and hydrocortisone	inpatient all-cause mortality	90 days
Niu JJ 2019	122/112	59	49.6%	3g IV QD	placebo	28-day mortality	28 days
