# Supplementary material for: Association of Oral or Intravenous Vitamin C Supplementation with Mortality: A Systematic Review and Meta-Analysis
Source: Nutrients. 2023 Apr 12;15(8):1848. doi: 10.3390/nu15081848 (PMC10146309; doi:10.3390/nu15081848)
Supplement: Supplementary file 1 [file nutrients-15-01848-s001.zip › supplemental Table S3 .pdf]

Supplemental Table S3 Ongoing trials of vitamin C supplementation on mortality.

**Registration**	**Health Condition(s) or Problem(s) studied**	**Location**	**Participants (age)**	**Dose**	**Outcomes**	**Current state**	**Results expected**
NCT04291508	Sepsis	Massachusetts General Hospital，USA	900 participants（Age ≥ 18 years）	50 mg/kg every six hours for 5 days (20 doses)	Days alive and free of organ support to day 28	Recruiting	July 2023
EUCTR2019-001086-32-DE	open-heart surgery with the use of cardiopulmonary bypass	RWTH Aachen University，Europe	30（Adults, Elderly）	Not reported	Include mortality	Ongoing	Not reported
EUCTR2020-001862-12-BE	sepsis or septic shock	University Hospitals Leuven, Europe	300（Adults, Elderly）	high doses of Vitamin C （ Not reported）	Secondary end point(s): 28-day mortality	Ongoing	Not reported
**CTRI/2020/10/028695**	**COVID-19**	**New Delhi, India**	**100（ Age ≥ 18 years，** ≤ 75 years）	**Oral 500mg（twice a day）**	**Assessment of symptoms**	Ongoing	Not reported
**ChiCTR2000030135**	**COVID-19**	**Xian，China**	**39** （（Age ≥ 18 years）	**high doses of Vitamin C（ Not reported）**	**Ventilation-free days; mortality;**	Ongoing	Not reported
**CTRI/2022/04/042012**	**head and neck cancer surgeries**	**New Delhi, India**	**60（ Age ≥ 18 years，** ≤ 65 years）	**Injection 1g a day for 4 days**	**Secondary Outcome may include mortality**	Ongoing	Not reported
**ChiCTR1900026084**	**Sepsis**	**Lanzhou，China**	**200**（Age ≥ 18 years）	**Vitamin C 1.5g q6h for 3 consecutive days**	**Mortality**	Ongoing	Not reported
NCT02516670	**Prostate Cancer**	**Johns Hopkins Sidney Kimmel Comprehensive Cancer Center，USA**	**50（ Male， Age ≥ 18 years）**	**ascorbic acid twice weekly（ Not reported）**	**serious adverse event：death**	Completed; recruitment completed	2022
**NCT04344184**	**COVID-19**	**Virginia Commonwealth University，USA**	**48（ Age ≥ 18 years ）**	**50 mg/kg intravenous every 6 hours for up to 96 hours**	**Secondary Outcome: Mortality by all causes**	Ongoing	Not reported
NCT03780933	**Acute Respiratory distress syndrome （ARDS）**	**Embaba Chest Hospitals Cairo, Egypt**	**40（ Age ≥ 18 years，** ≤ **70 years）**	**10 g IV**	**Assessment of Improvement in patient's mortality rate**	recruitment completed	Not reported

**IRCT20190312043030N1**	**severe pneumonia**	**Tabriz University of Medical Science, Iran**	**80 (Age ≥ 18 years，** ≤ 90 years）	**50mg/kg/day**	**Mortality; Mechanical ventilation free days**	recruitment completed	Not reported
**CTRI/2019/07/020370**	**multiple trauma**	**PONDICHERRY, India**	**136 (Age ≥ 18 years，≤ 90 years）**	**2 mg in 500 ml NS as continuous infusion for 3 days**	**30 days mortality**	recruitment completed	Not reported
**SLCTR/2017/028**	**Dengue fever**	**Teaching Hospital, Karapitiya, Sri Lanka.**	**60 (Age ≥12 years， ≤ 70 years）**	**Oral 1000mg bid (twice daily) for 5 days**	**Secondary bacterial infection may include mortality**	recruitment completed	Not reported
**NCT04494451**	**Critically Ill Cirrhotics with Multidrug-resistant Bacterial Infections**	**Institute of Liver and Biliary Sciences, India**	**100 (Age ≥ 18 years，≤ 70 years）**	**(25 mg/kg or max. 1.5 gram every 6 hourly) for maximum 5 day**	**Mortality in both groups**	Not yet recruiting	Not reported
**CTRI/2020/09/027527**	**Critically ill cirrhotics with MDR infections**	**Institute of Liver and Biliary Sciences, India**	**100 (Age ≥ 18 years，≤ 70 years）**	**(25 mg per kg or max. 1.5 gram every 6 hourly)5 Days**	**Mortality in both groups**	Not yet recruiting	Not reported
**ACTRN12620000651987**	**severe infection Septic shock**	**Austin Hospital, Australia**	**30(Age ≥ 18 years)**	**60 grams in a day**	**Hospital mortality**	Recruiting	Not reported
**NCT04029675**	**Sepsis**	**Cairo, EL Abbasia, Egypt**	**40 (Age ≥ 18 years， ≤ 65 years）**	**1.5 gm intravenous for 4 days or until ICU discharge.**	**28-day Mortality rate**	recruitment completed	Not reported
**NCT04088591**	**liver disease With Multidrug-resistant Bacterial Infections.**	**DELHI , India**	**200 (Age ≥ 18 years，≤ 80 years）**	**25 mg per kg or max. 1.5 gram every 6 hourly**	**Mortality in both groups, time point: Day 28**	Ongoing	Not reported
**NCT03175341**	**Breast Cancer**	**Belgium, Europe**	**30 (Age ≥ 18 years)**	**1,5 g on Day 1 followed by 0,75g on Day 2-4 at each chemotherapy cycle**	**Adverse Events may include mortality**	Not yet recruiting	Not reported
**IRCT20200516047468N1**	**COVID-19**	**Tehran, Iran**	**50 (Age ≥ 18 years)**	**2 grams every 6 hours for 5 days**	**Secondary outcomes: mortality rate**	Ongoing	Not reported
**NCT02969681**	**Colorectal Neoplasms**	**Guangzhou, Guangdong,**	**428 (18 Years to 75 Years)**	**(1.5g/kg/day) Day 1-3, every 2 weeks**	**Secondary endpoints**	Ongoing	Not reported

		China			including overall survival		
NCT03015675	Gastric Cancer	Sun Yat-sen University, China	200 (Age ≥ 18 years)	20g/day, Day1-3, every 2 weeks	Overall Survival	Recruiting	Not reported
NCT04401150	COVID-19	Sherbrooke, Quebec, Canada	800 (18 Years and older)	50 mg/kg intravenously every 6 hours for 96 hours (16 doses).	Death or persistent organ dysfunction	Suspended	December 2022
NCT04404387	Septic, Acute Respiratory Distress Syndrome	France, Europe	800 (18 Years and older)	50 mg/kg every 6 hours for 96 hours	Mortality at 28 days and 6 months	Recruiting	July 2024
NCT01905150	Pancreatic Cancer	New York, United States	34 (18 Years and older)	High dose of Vitamin C, not reported.	12-month survival rate	Recruiting	Not reported
ChiCTR1900022022	Acute Pancreatitis	Shanghai, China	418 (18 Years to 80 Years)	200 mg/kg/h for 7 days	28 days mortality	Pending	Not reported
CTRI/2020/12/029688	traumatic brain injury	Banaras Hindu university, India	60 (age > 18 years and < 65 years)	500mg(5ml) iv tid OR 10 g on first day and repeated on 4th day, followed by 4g/day for remaining 3 days	Effect on mortality and infection	Not Yet Recruiting	Not reported
CTRI/2018/05/013994	Sepsis	TAMIL NADU, India	60 (age > 18 years)	1.5 g every 6 hours for 4 days (24 doses)	In hospital all-cause mortality	recruitment completed	Not reported
NCT01090895	Myocardial infarction	Roma, Italy	80 (18 Years to 85 Years)	(1 g) 10 minutes before percutaneous coronary intervention.	Cardiovascular Death	Recruiting	No Results Posted
ACTRN12619000256178	Community Acquired Pneumonia	Wellington, New Zealand	140 (age > 18 years)	Intravenous infusion of 2.5 g /8 hours at maximum of 7 days, 7 days of oral vitamin C at a dose of 1 g tid.	All-cause mortality	Not yet recruiting	Not reported
NCT03509662	Cardiac Arrest	Amsterdam UMC, Netherland	270 (age > 18 years)	Vitamin C - 3 gr/day or 10 gr/day	Mortality at 30 days and 180 days	Recruiting	Not reported
NCT05029037	COVID-19	Mexico	160 (18 Years to 80 Years)	Two (2) High doses of intravenous vitamin C for seven (7) days.	Mortality	Not yet recruiting	2022
NCT05194189	Sepsis Septic Shock	Guangzhou, China	152 （18 Years to 80 Years）	12 g (48 ml) every 12 h for 4 days or at ICU discharge	28-day all-cause mortality	Recruiting	January 4, 2024
