# Supplementary material for: Association of Oral or Intravenous Vitamin C Supplementation with Mortality: A Systematic Review and Meta-Analysis
Source: Nutrients. 2023 Apr 12;15(8):1848. doi: 10.3390/nu15081848 (PMC10146309; doi:10.3390/nu15081848)
Supplement: Supplementary file 1 [file nutrients-15-01848-s001.zip › supplemental Table S4.pdf]

Supplemental Table S4 sensitivity analyses

Sensitivity analyses	Risk Ratio, 95% CI	I^2	p
Excluding studies with high or unknow risk of bias.	0.74(0.57-0.96)	45%	0.02
Excluding trails before the year of 2000.	0.94(0.84-1.05)	36%	0.01
Excluding the trail with the largest number of participants.	0.69(0.56-0.85)	32%	0.03
Excluding trails with follow-up time longer than 1 month.	0.60(0.47-0.78)	24%	0.10
Excluding trials with the largest heterogeneity of each subgroup.	0.72(059-0.88)	36%	0.02
Excluding studies with high or unknow risk of bias of the different domains.			
Sequence generation.	0.88(0.81-1.01)	20%	0.20
Allocation concealment.	0.78(0.89-1.09)	25%	0.30
Blinding of patients and personnel.	0.91(0.79-1.01)	31%	0.21
Blinding of outcome assessors.	0.92(0.88-1.00)	28%	0.34
