# Supplementary figures and images for: Association of Oral or Intravenous Vitamin C Supplementation with Mortality: A Systematic Review and Meta-Analysis
Source: Nutrients. 2023 Apr 12;15(8):1848. doi: 10.3390/nu15081848 (PMC10146309; doi:10.3390/nu15081848)

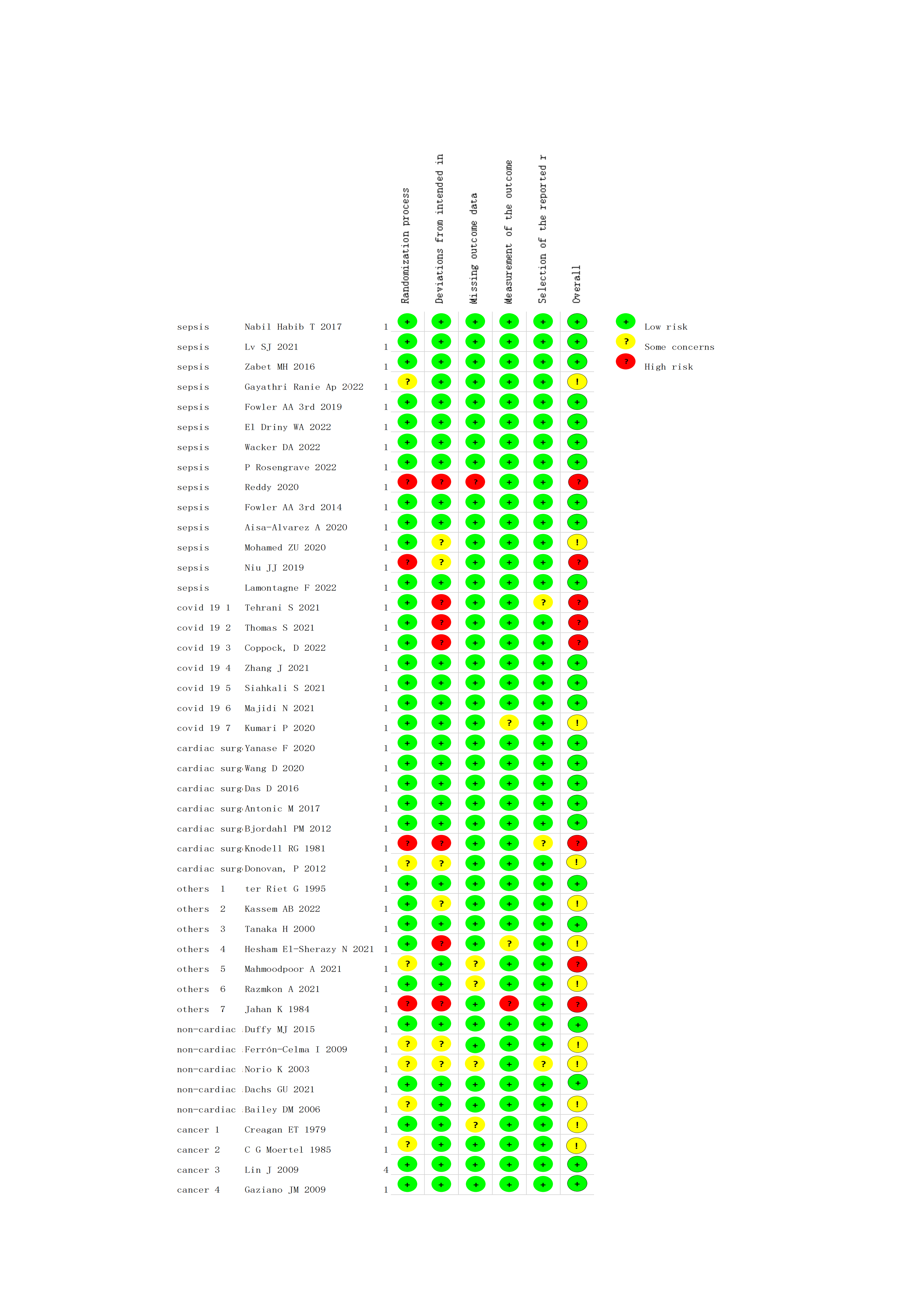

Supplement: Supplementary file 1 [file nutrients-15-01848-s001.zip › supplemental figure S1.png]

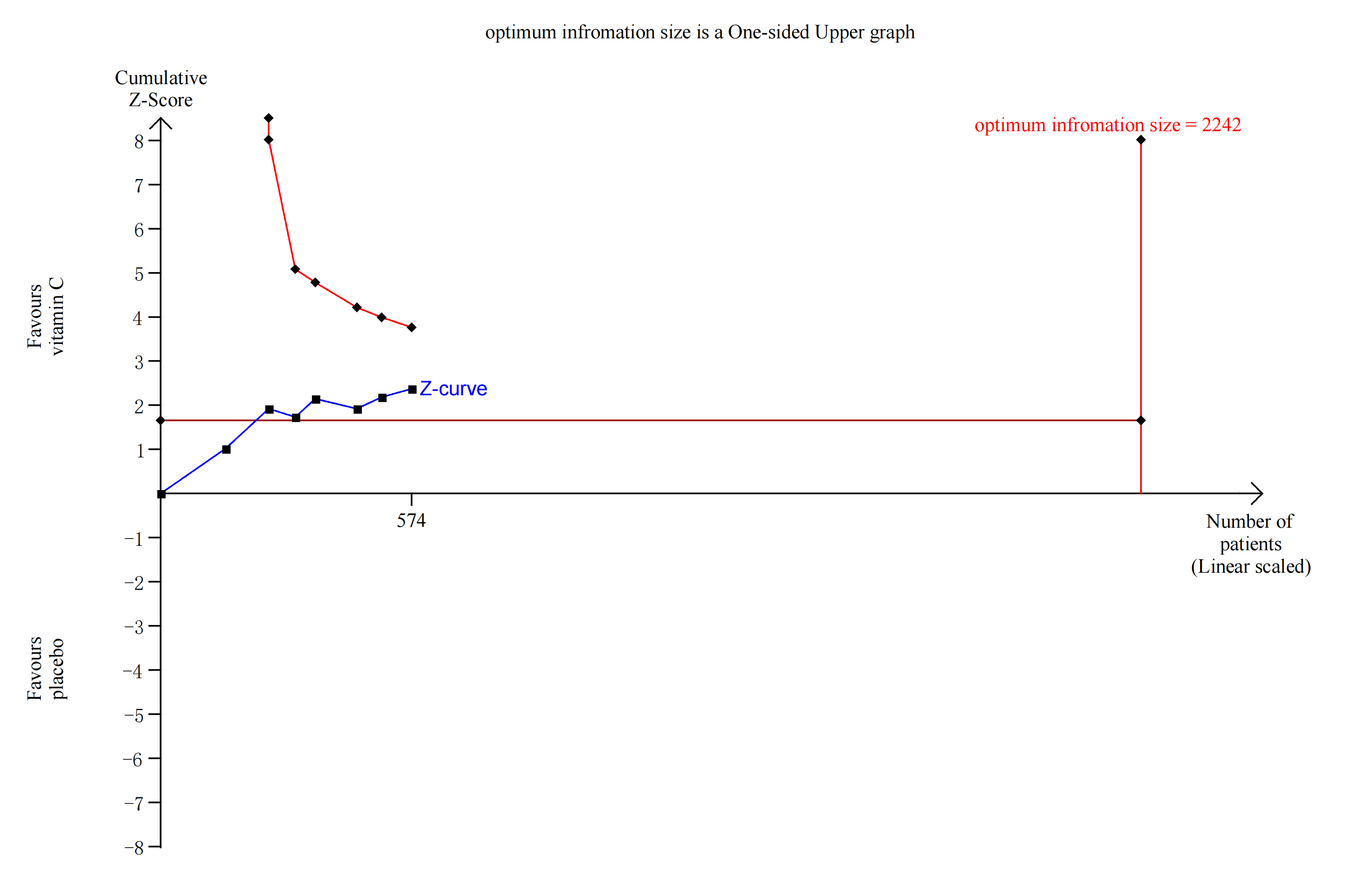

Supplement: Supplementary file 1 [file nutrients-15-01848-s001.zip › supplemental figure S10.png]

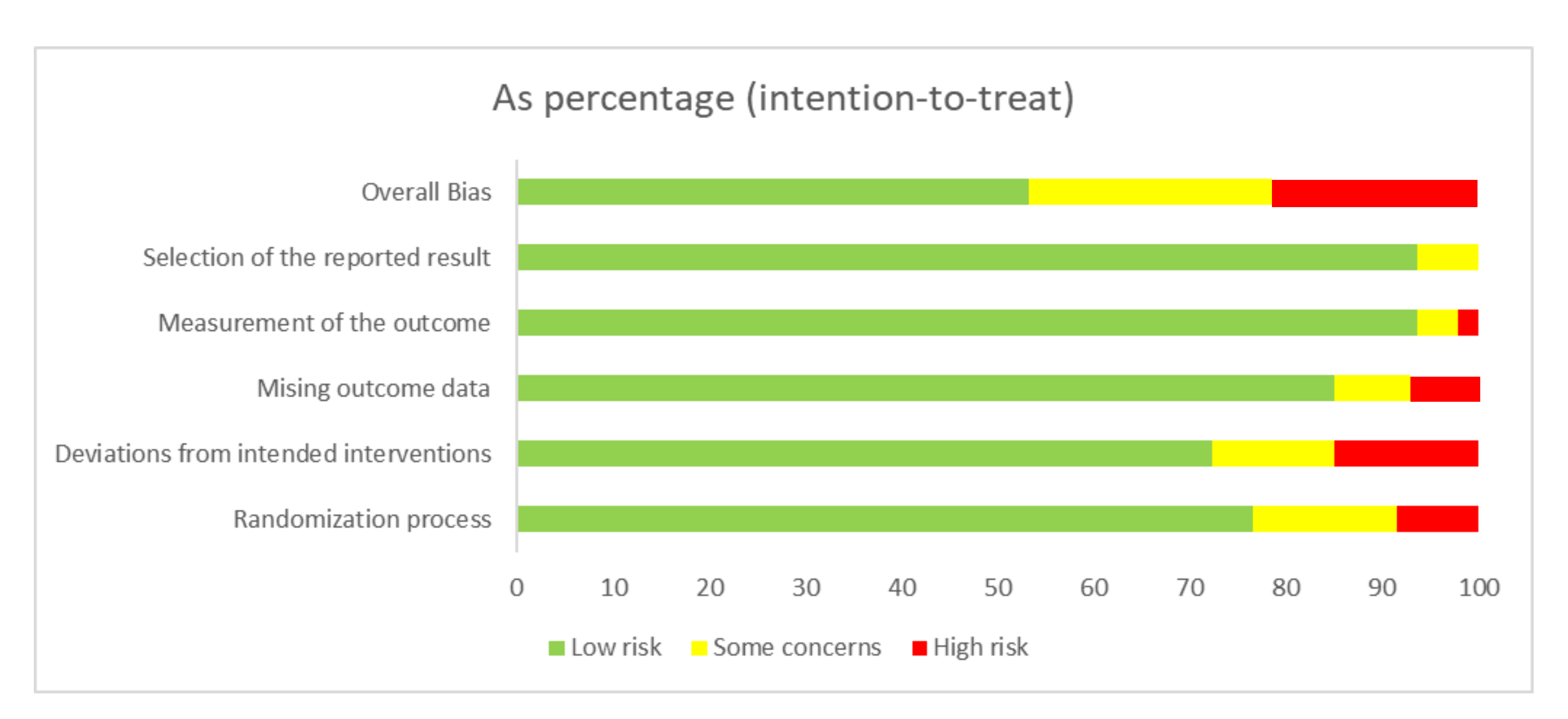

Supplement: Supplementary file 1 [file nutrients-15-01848-s001.zip › supplemental figure S2.png]

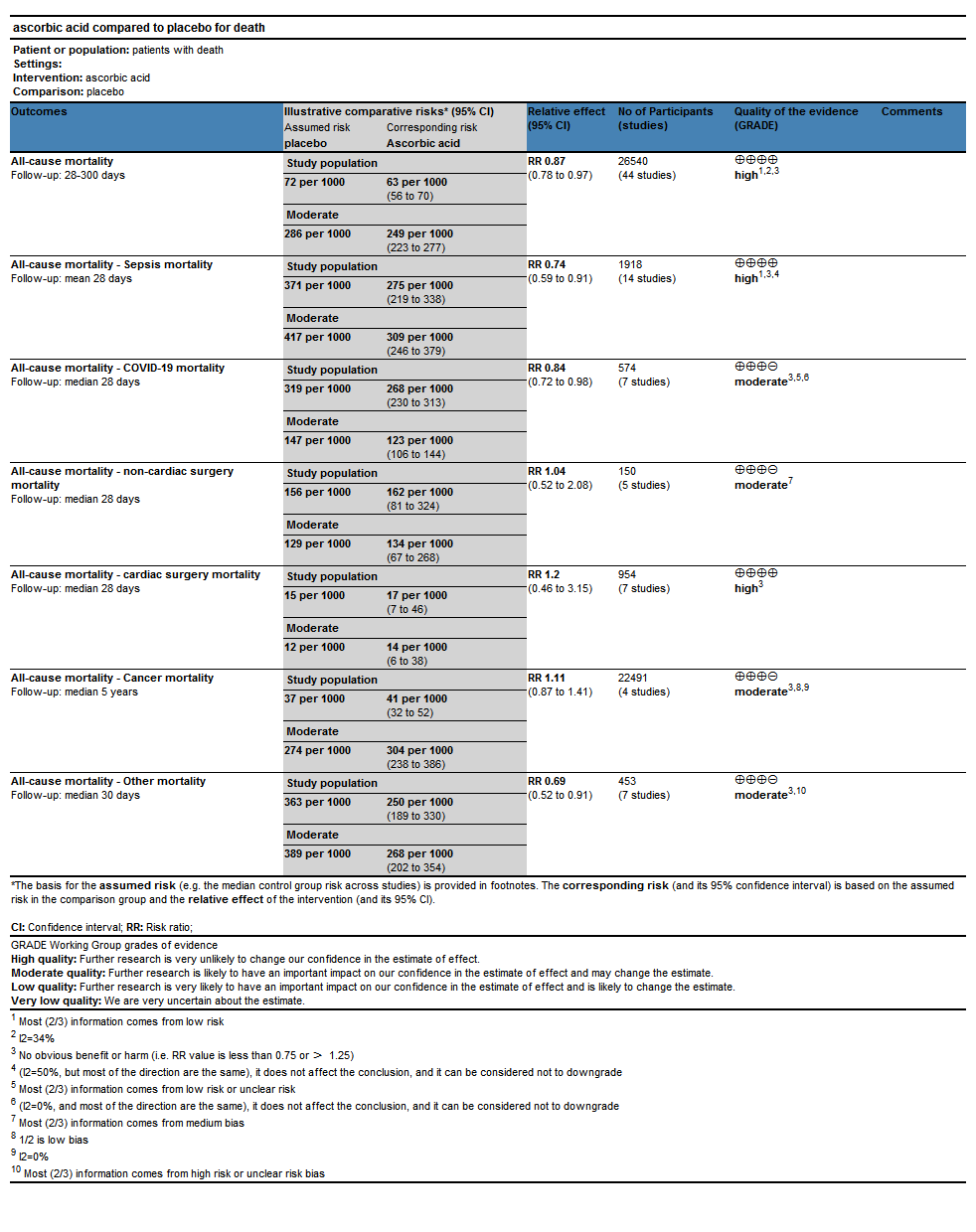

Supplement: Supplementary file 1 [file nutrients-15-01848-s001.zip › supplemental figure S3.png]

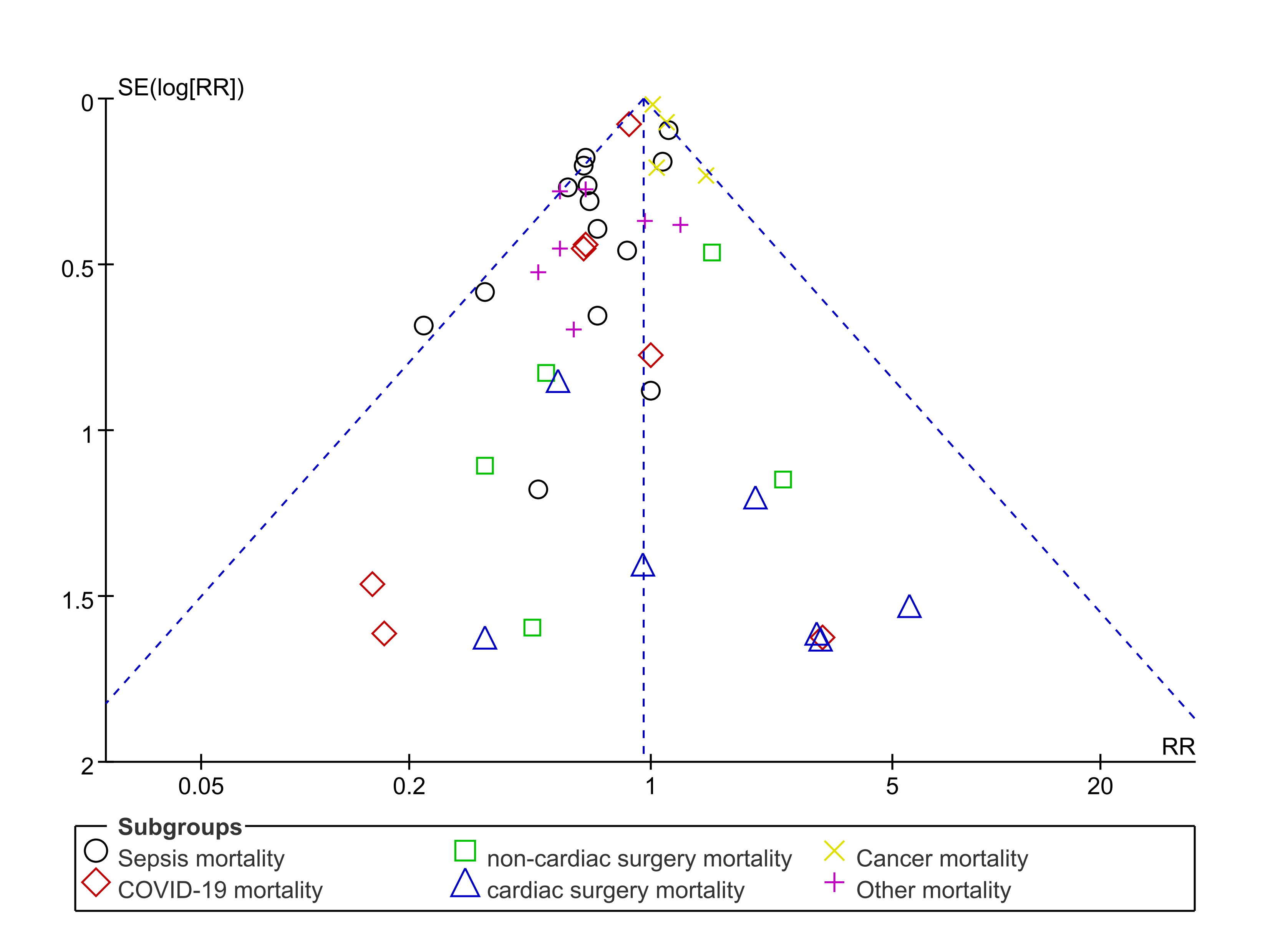

Supplement: Supplementary file 1 [file nutrients-15-01848-s001.zip › supplemental figure S4.png]

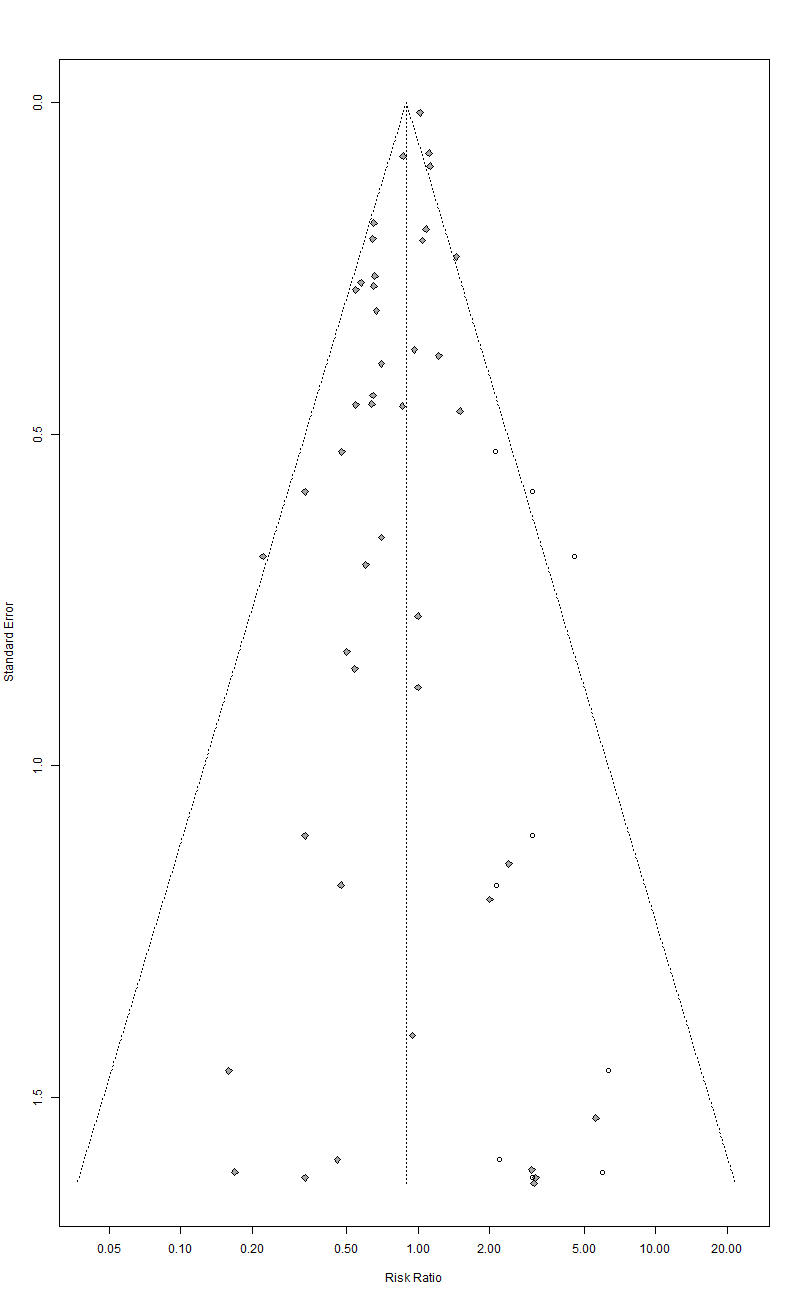

Supplement: Supplementary file 1 [file nutrients-15-01848-s001.zip › supplemental figure S5.png]

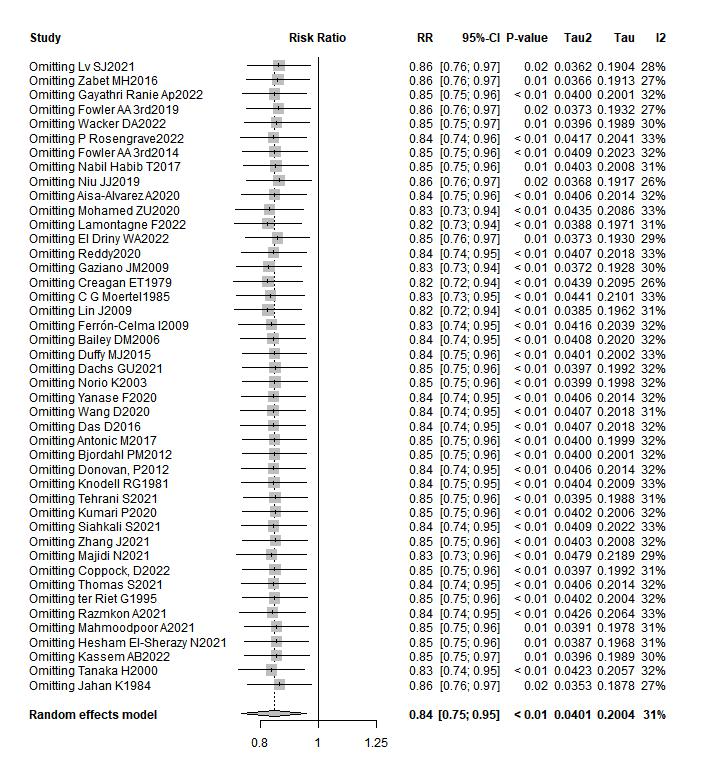

Supplement: Supplementary file 1 [file nutrients-15-01848-s001.zip › supplemental figure S6.png]

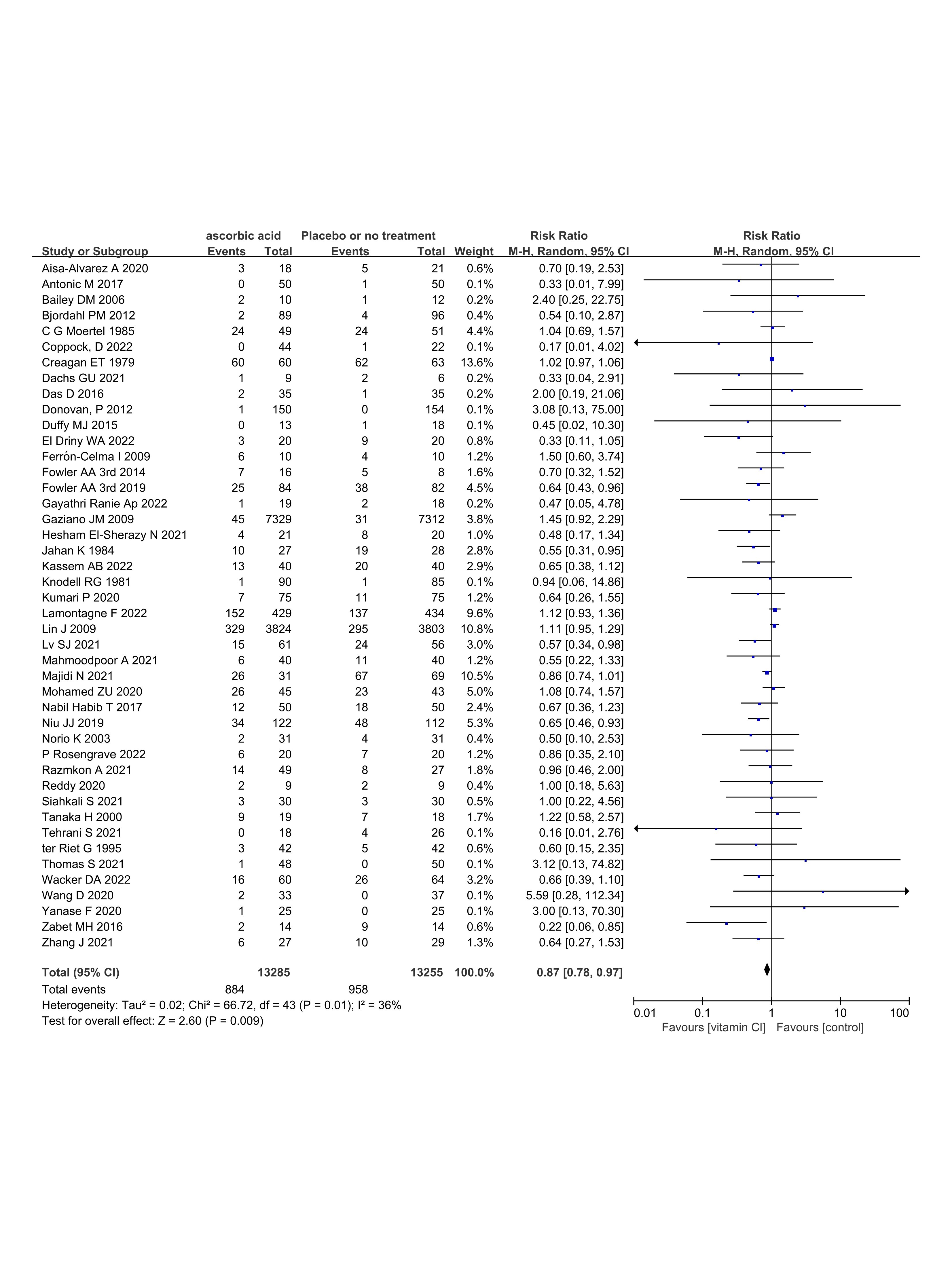

Supplement: Supplementary file 1 [file nutrients-15-01848-s001.zip › supplemental figure S7.png]

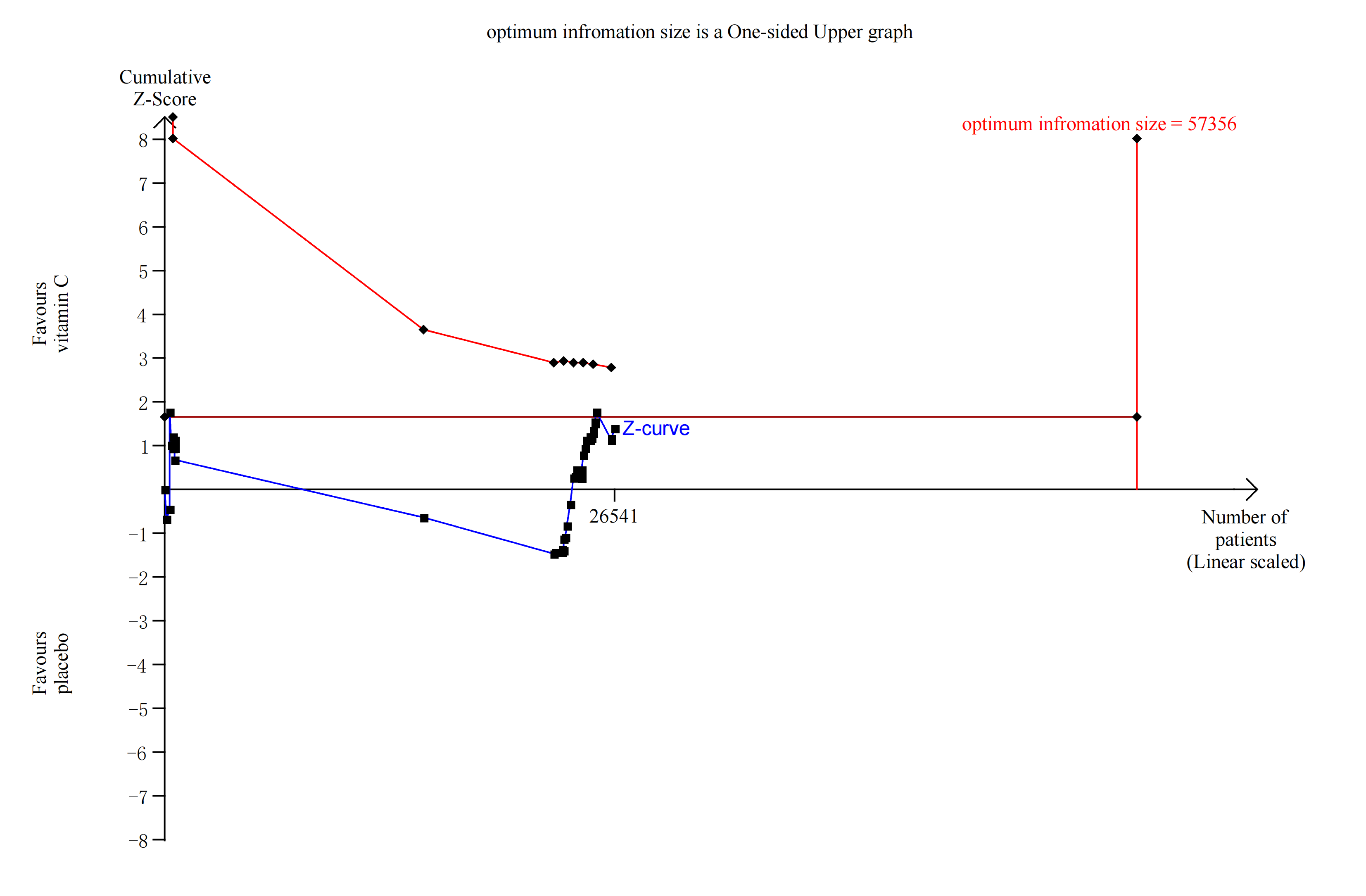

Supplement: Supplementary file 1 [file nutrients-15-01848-s001.zip › supplemental figure S8.png]

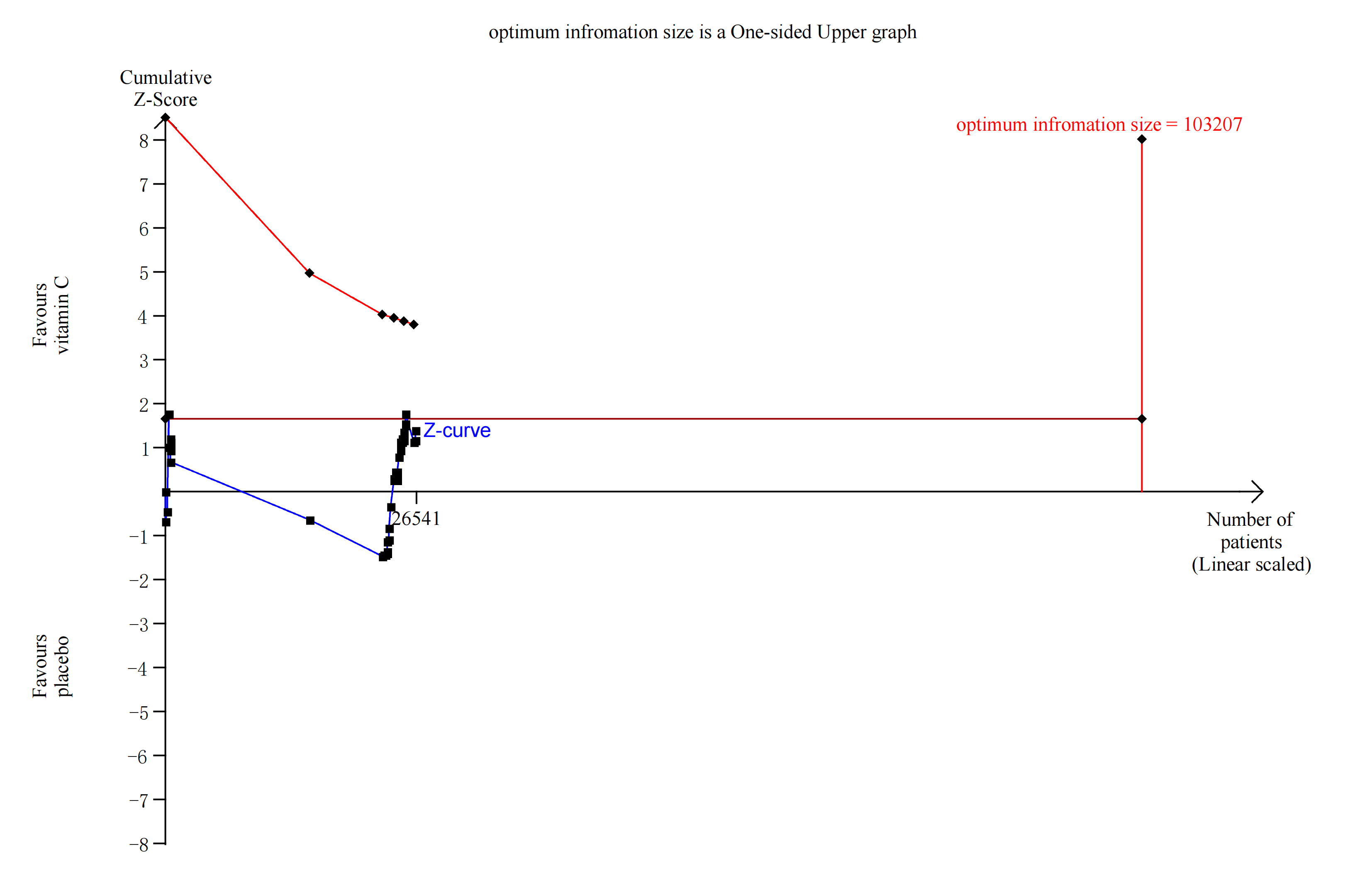

Supplement: Supplementary file 1 [file nutrients-15-01848-s001.zip › supplemental figure S9.png]
